# Supplementary material for: Trophoblast stem cell-based organoid models of the human placental barrier
Source: Nat Commun. 2024 Feb 8;15:962. doi: 10.1038/s41467-024-45279-y (PMC10853531; doi:10.1038/s41467-024-45279-y)
Supplement: Supplementary file 1 — Supplementary Information [file 41467_2024_45279_MOESM1_ESM.pdf]

Trophoblast stem cell-based organoid models of the human placental barrier

Takeshi Hori[1], Hiroaki Okae[2,3], Shun Shibata[2], Norio Kobayashi[2,4], Eri H Kobayashi[2], Akira Oike[2,3], Asato Sekiya[3], Takahiro Arima[2], and Hirokazu Kaji[1]*

[1] Department of Diagnostic and Therapeutic Systems Engineering, Institute of Biomaterials and Bioengineering (IBB), Tokyo Medical and Dental University (TMDU), 2-3-10 Kanda-Surugadai, Chiyoda-ku, Tokyo 101-0062, Japan.

[2] Department of Informative Genetics, Environment and Genome Research Center, Tohoku University Graduate School of Medicine, 2-1 Seiryo-cho, Aoba-ku, Sendai, 980-8575, Japan.

[3] Department of Trophoblast Research, Institute of Molecular Embryology and Genetics, Kumamoto University, Kumamoto, 862-0973, Japan.

[4] Department of Mechanical Engineering, University of Michigan, Ann Arbor, MI 48109, USA

*E-mail: kaji.bmc@tmd.ac.jp

Fig. S1

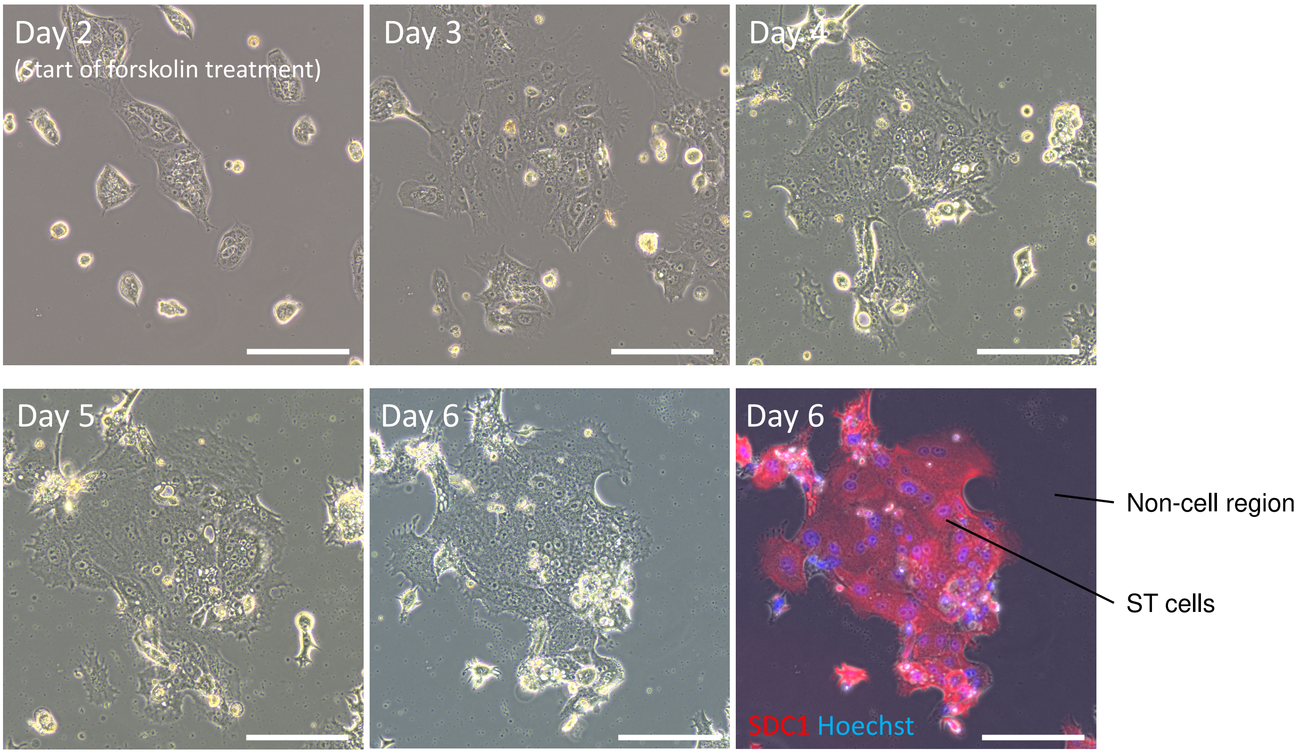
Day 2
(Start of forskolin treatment)
Day 3
Day 4
Day 5
Day 6
Day 6
SDC1 Hoechst
Non-cell region
ST cells

Fig. S1. Differentiation of ST cells on a culture plate.

A 6-well plate was coated with 10 μg/mL collagen IV at 37ºC for >1.5 h and washed with PBS(-). TS cells were then seeded at 0.5 x 10^5 cells/well to the wells and cultured with TS medium without iMatrix-511. After 2 days, culture media were changed to ST medium (DMEM/F12 supplemented with 0.3% BSA, 50 units/mL penicillin, 50 μg/mL streptomycin, 1% ITS-X, 4% KSR, 2.5 μM Y27632, and 2 μM forskolin). On Day 4, the medium was changed. On day 6, cells were fixed with 4% PFA for 10 min, treated with 0.3% Triton X-100 for 5 min, and stained with an antibody for SDC1. Scale bars indicate 200 μm.

Fig. S2

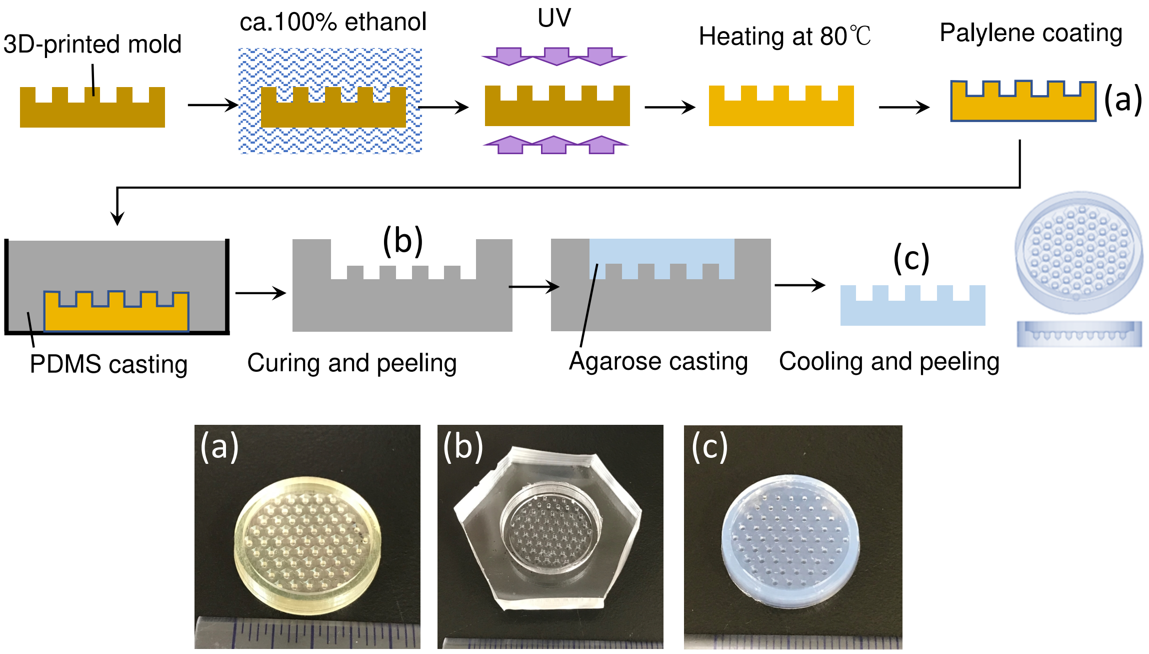
3D-printed mold
ca.100% ethanol
UV
Heating at 80℃
Palylene coating
(a)
(b)
(c)
PDMS casting
Curing and peeling
Agarose casting
Cooling and peeling
(a)
(b)
(c)

Fig. S2. Fabrication of agarose microwell plates.

A microwell plate was designed using SolidWorks 2019 (Dassault Systèmes SolidWorks Corporation) and Materialise MiniMagics23.5 (Materialise). Agarose microwell plates were fabricated using the procedure described in Methods. (a) A 3D-printed mold. (b) A PDMS mold. (c) An agarose microwell plates.

Fig. S3

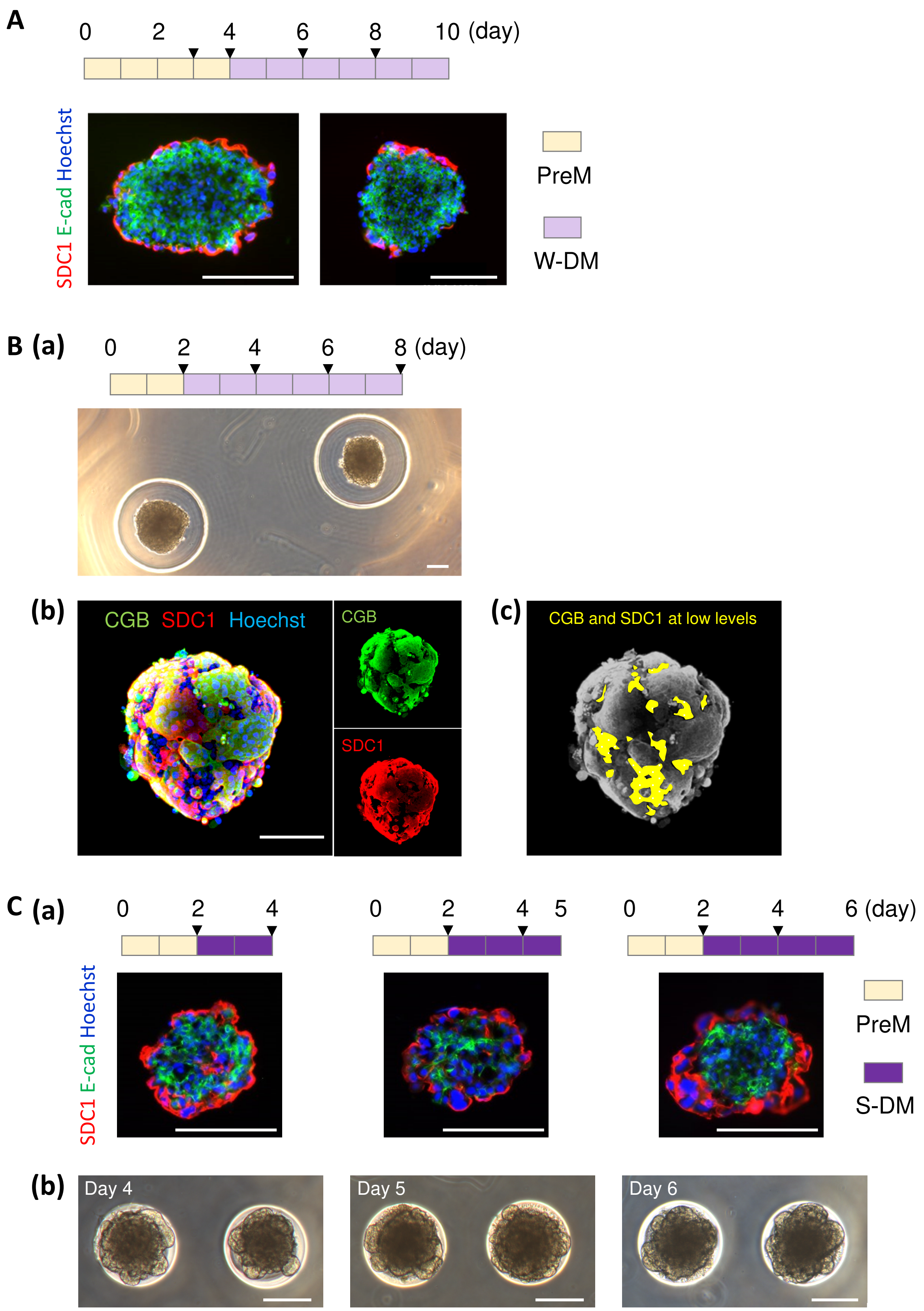
A
0 2 4 6 8 10 (day)
SDC1 E-cad Hoechst
PreM
W-DM
B (a)
0 2 4 6 8 (day)
(b)
CGB SDC1 Hoechst
CGB
SDC1
(c)
CGB and SDC1 at low levels
C (a)
0 2 4
0 2 4 5
0 2 4 6 (day)
SDC1 E-cad Hoechst
PreM
S-DM
(b)
Day 4
Day 5
Day 6

Fig. S3. Treatment of TS cells with two kinds of culture medium.
(A-C) TS cells were seeded into agarose microwell plates and cultured according to each culture schedule. Scale bars indicate 200 μm. Triangular marks indicate medium exchange.
(A) Immunostaining of SDC1 and E-cadherin and staining of nuclei with Hoechst. Frozen sections samples were prepared and imaged using BZ-X800/810.
(B) A representative image of trophoblast organoids (a), immunostaining of CGB and SDC1 (b), and the surface region highlighted in yellow showing lower expression of both CGB and SDC1 proteins (c). (b and c) Images were obtained by confocal microscopy.
(C) Immunostaining of SDC1 and E-cadherin and staining of nuclei with Hoechst(a). Frozen sections samples were prepared and imaged using BZ-X800/810. Images of spherical trophoblast organoids were taken using a phase contrast microscope (b)(CKX53, Olympus).

Fig. S4

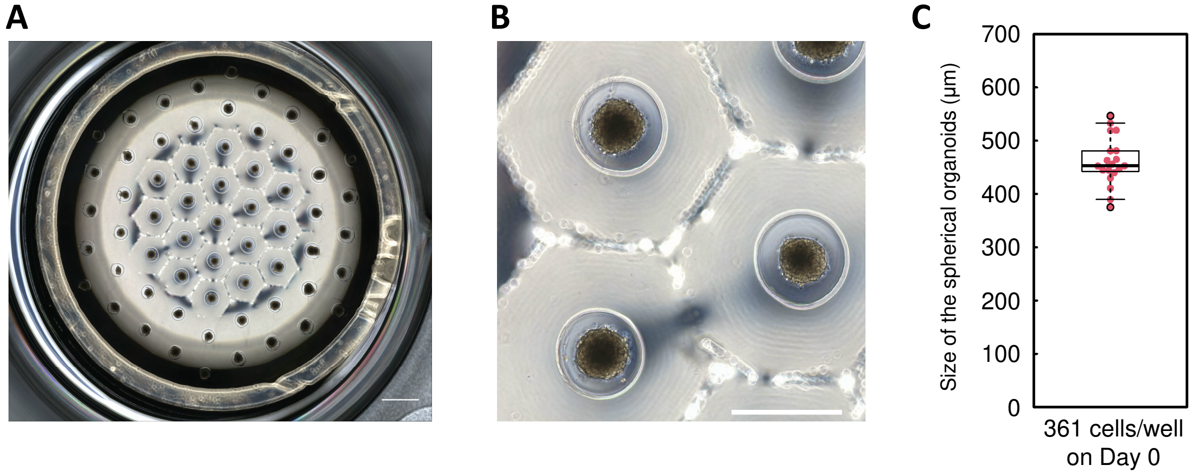

A
B
C
700
600
500
400
300
200
100
0
Size of the spherical organoids (µm)
361 cells/well
on Day 0

Fig. S4. Size of the spherical trophoblast organoids in the 61-well agarose plate

(A) The spherical organoids were generated using microwell plates that were designed in Fig. S2 and fixed with 4% PFA on Day 8. A whole image of a microwell plate with the spherical organoids was created by taking and combining 100 images using BZ-X800/810. The scale bars indicate 2 mm.

(B) A partially magnified image of Fig. S4(A).

(C) Size (diameter) of the spherical organoids was measured using ImageJ version 1.47t (NIH). The organoids (N=19) positioned and focused in the central area were measured.

Images were taken using BZ-X800/810.

The scale bars indicate 2000 μm (A) or 1000 μm (B).

Fig. S5

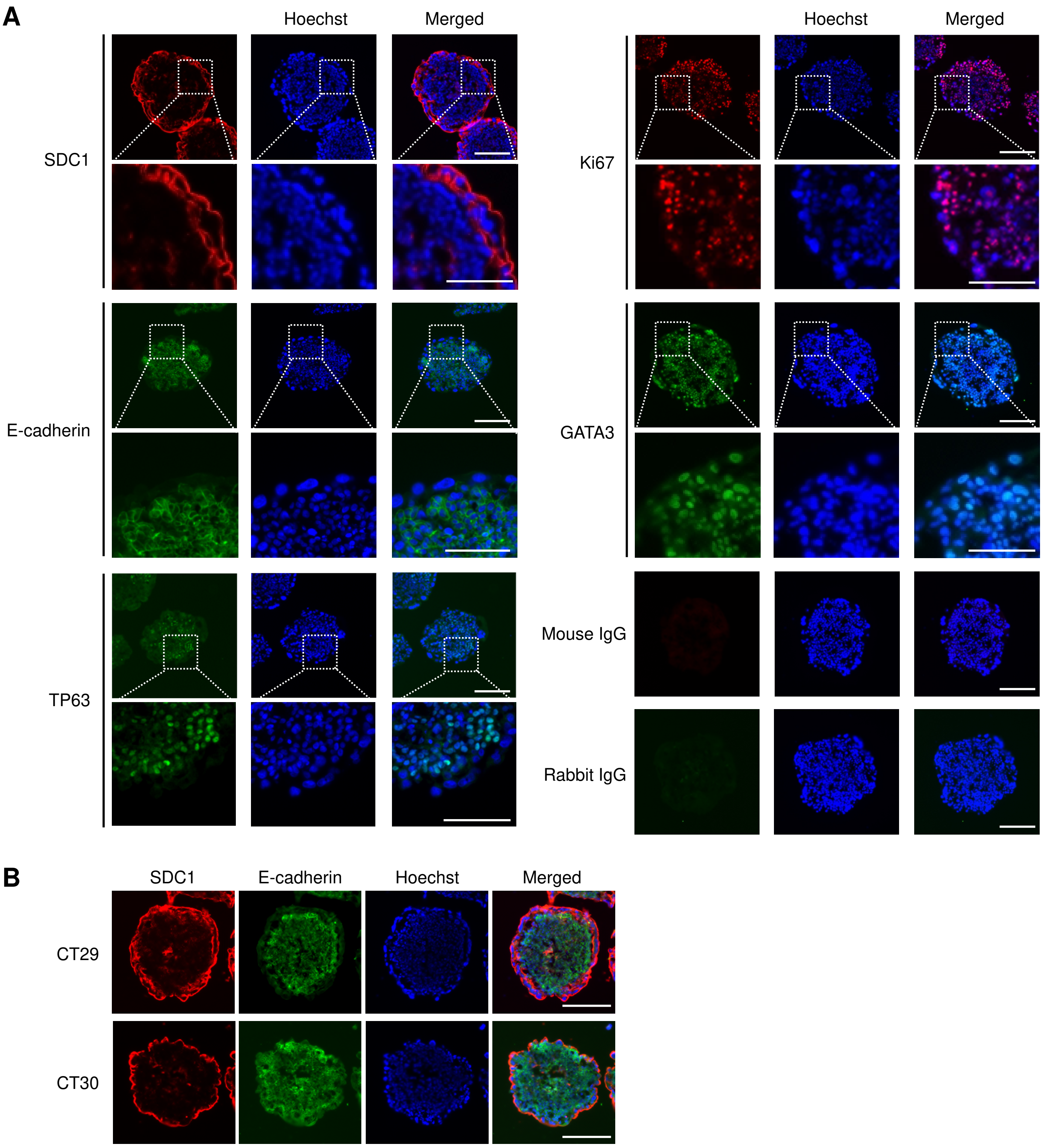
A
Hoechst
Merged
SDC1
E-cadherin
TP63
Hoechst
Merged
Ki67
GATA3
Mouse IgG
Rabbit IgG
B
SDC1
E-cadherin
Hoechst
Merged
CT29
CT30

Fig. S5. Immunostaining analysis of trophoblast organoids.

(A) Apical-out spherical trophoblast organoids were generated from a TS cell line CT27 by the same procedure as described in Figure 1A. Immunostaining analysis with antibodies for syndecan 1 (SDC1), E-cadherin, TP63, Ki67, GATA3, normal mouse IgG (negative control), or normal rabbit IgG (negative control) was conducted for cross-sections of the organoids. The scale bars indicate 200 μm or 100 μm (magnified images).

(B) Expression of SDC1 and E-cadherin in other TS cell lines, CT29 and CT30. CT29 and CT30 were immunostained as well as the CT27 cell line that was mostly used in the present study. The scale bar indicates 200 μm. Frozen sections samples were prepared and imaged using BZ-X800/810. Section samples were prepared from the organoids of day 8.

Fig. S6

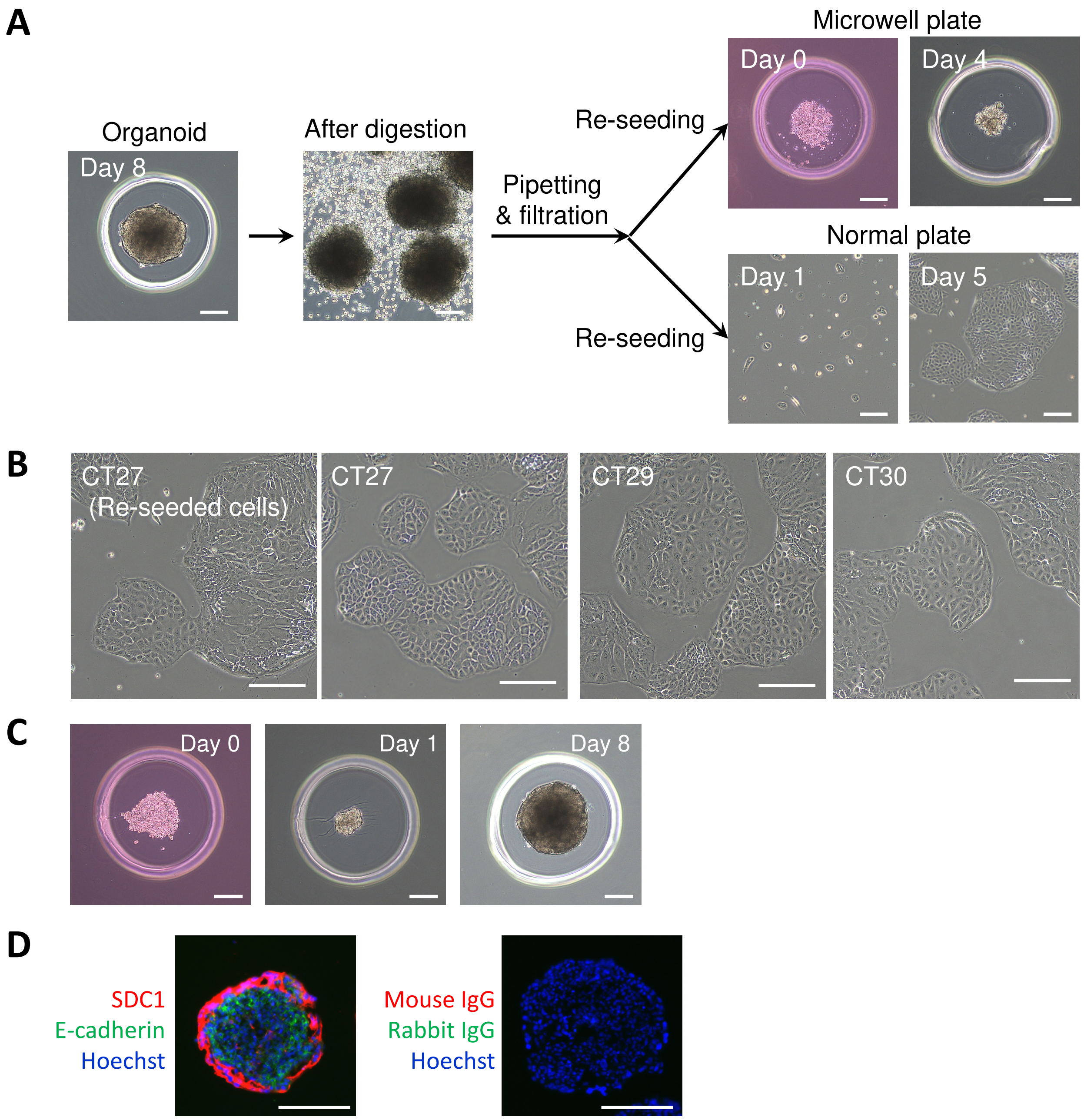
A
Organoid
Day 8
After digestion
Pipetting
& filtration
Re-seeding
Microwell plate
Day 0
Day 4
Re-seeding
Normal plate
Day 1
Day 5
B
CT27
(Re-seeded cells)
CT27
CT29
CT30
C
Day 0
Day 1
Day 8
D
SDC1
E-cadherin
Hoechst
Mouse IgG
Rabbit IgG
Hoechst

Fig. S6. Passage of apical-out spherical trophoblast organoids

(A) The spherical trophoblast organoids were generated from CT27 by the same procedure as described in Fig. 1A and then digested with TrypL Express Enzyme (1X) for 40 min at 37°C with 5% CO_2. After pipetting, cells were filtrated with 20 μm pluriStrainer (Cat# 43-50020-01, pluriSelect) and re-seeded to the agarose microwell plates or wells of 6-well plates. Cells in the microwell plates were cultured with the medium for generation of apical-out spherical trophoblast organoids. Cells in wells were cultured with TS medium.

(B) The shapes of the re-seeded cells were compared with the normally cultured TS cell lines (CT27, CT29, and CT30).

(C) The re-seeded cells on the wells were harvested and seeded to agarose microwell plates and cultured to generate the spherical organoids.

(D) Frozen sections samples were prepared, stained with given antibodies, and imaged.

Images of spherical trophoblast organoids were taken using a phase contrast microscope CKX53 (A-C) or BZ-X800/810 (D). The scale bars indicate 200 μm.

Fig. S7

A

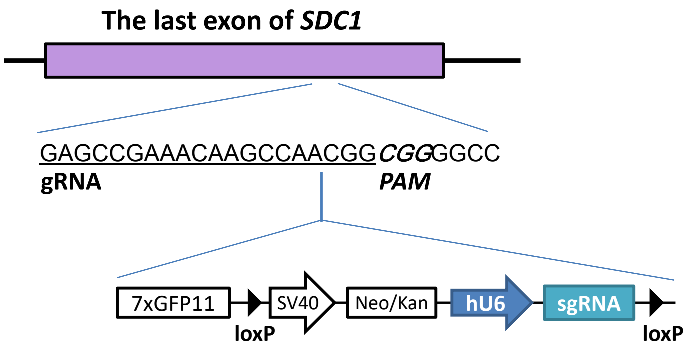

The last exon of SDC1
GAGCCGAAACAAGCCAACGGCGGGGCC
gRNA
PAM
7xGFP11
loxP
SV40
Neo/Kan
hU6
sgRNA
loxP

B

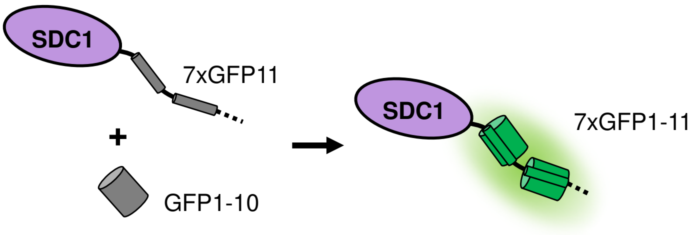

SDC1
7xGFP11
+
GFP1-10
SDC1
7xGFP1-11

Fig. S7. Split fluorescent protein systems for SDC1-GFP proteins

(A) Insertion of a DNA fragment for 7xGFP11 into the last exon of the SDC1 gene.

(B) A schematic illustration of the split fluorescent protein systems with 7xGFP11 and GFP1-10.

Fig. S8

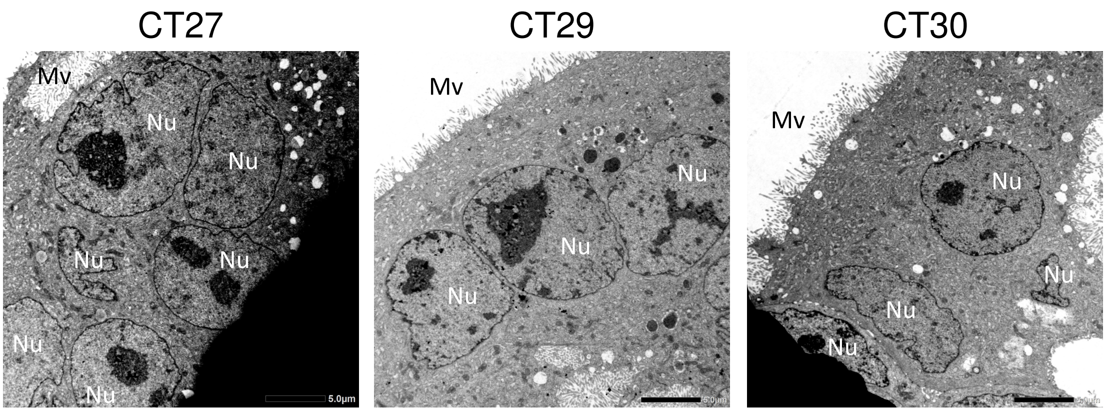

Fig. S8. TEM images of the spherical trophoblast organoids of each TS cell line (CT27, CT29, or CT30)

The surfaces of the spherical organoids were analyzed using a transmission electron microscope (JEM-1400Flash, JEOL, Japan). The scale bars indicate 5 μm. Images from the organoid models of day 8. Nu, nucleus. Mv, microvilli on the surface of the organoids.

Fig. S9

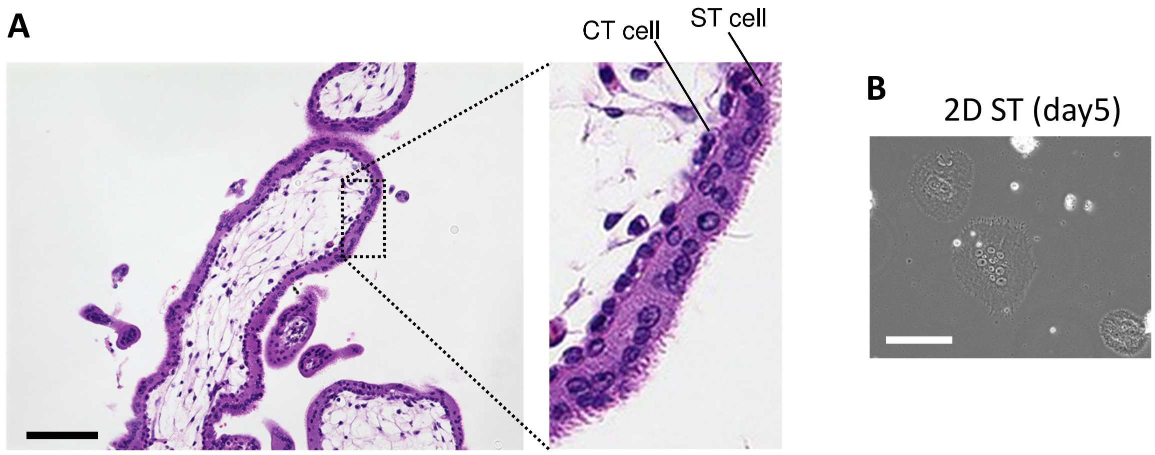

Fig. S9. Human placental villi and ST cells in 2D cultures.

(A) H&E staining of first trimester human placental villi. Microvilli are abundant on the apical surface of ST cells. The scale bar indicates 100 μm.

(B) ST cells in 2D cultures. TS cells were seeded into wells of a 6-well plate coated with collagen IV. Cells were differentiated into ST cells in a ST(2D) medium (DMEM/F12 supplemented with 0.3% BSA, 50 units/mL penicillin, 50 μg/mL streptomycin, 1% ITS-X, 4% KSR, 2.5 μM Y27632, and 2 μM Forskolin) for 5 days. The scale bar indicates 200 μm.

Fig. S10

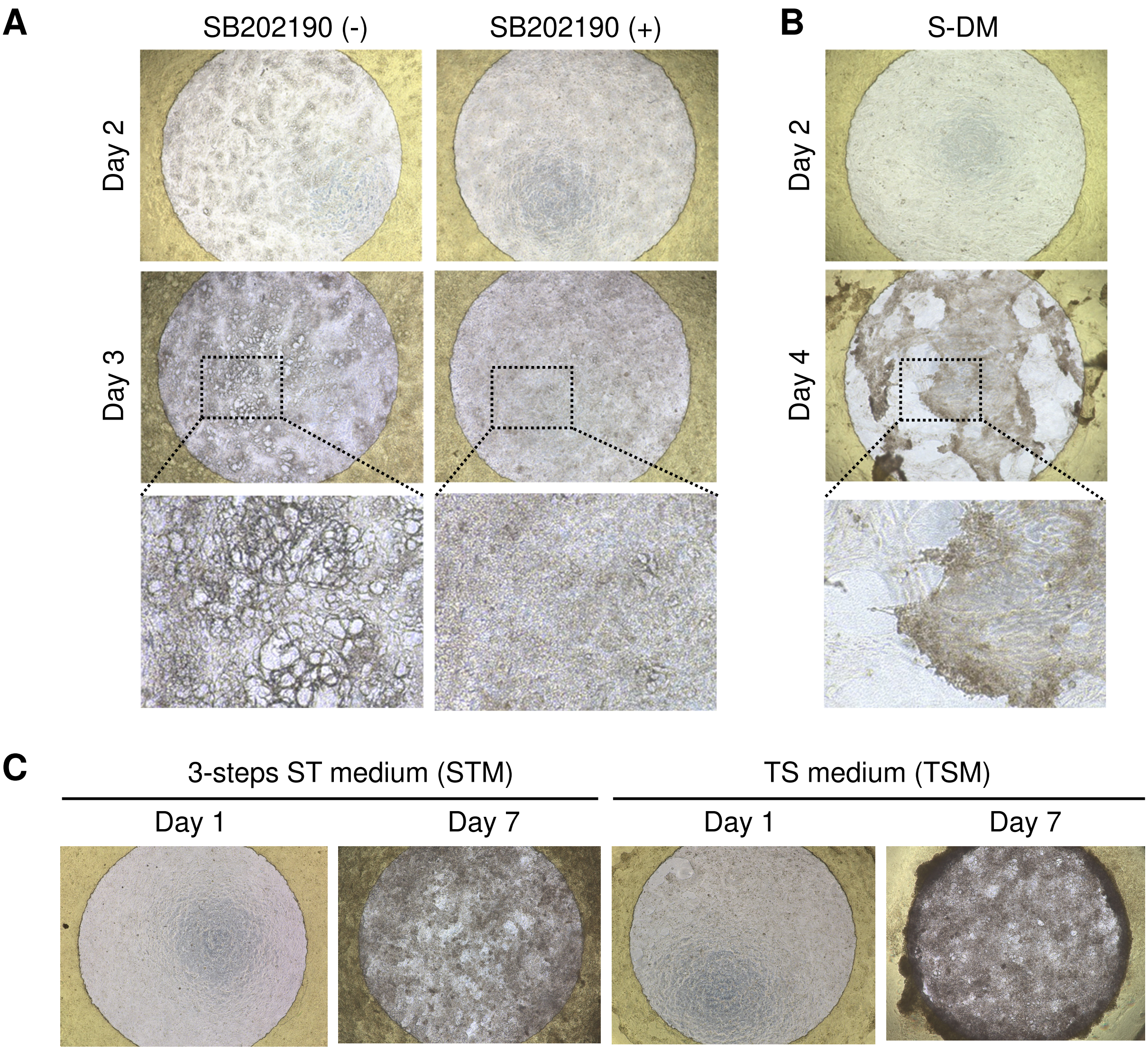
A
SB202190 (-)
SB202190 (+)
Day 2
Day 3
B
S-DM
Day 2
Day 4
C
3-steps ST medium (STM)
TS medium (TSM)
Day 1
Day 7
Day 1
Day 7

Fig. S10. Consideration for the generation of the ST barrier models.

(A) Effects of SB202190 on ST barrier formation. TS cells were seeded on the collagen membrane coated with Matrigel and cultured in PreM with or without SB202190 for 3 days.

(B) Effects of fetal bovine serum (FBS) on ST barrier formation. TS cells were cultured in S-DM for 4 days.

(C) Comparison of TS cells cultured in STM and TSM. Representative images for an ST barrier model and a control TS model. ST barrier models were generated under the culture condition with three kinds of medium (PreM, W-DM, and S-DM) shown in Fig. 3D(a). STM, Three kinds of medium for ST barrier formation. TSM, trophoblast stem cell medium.

Images were taken using BZ-X800/810.

Fig. S11

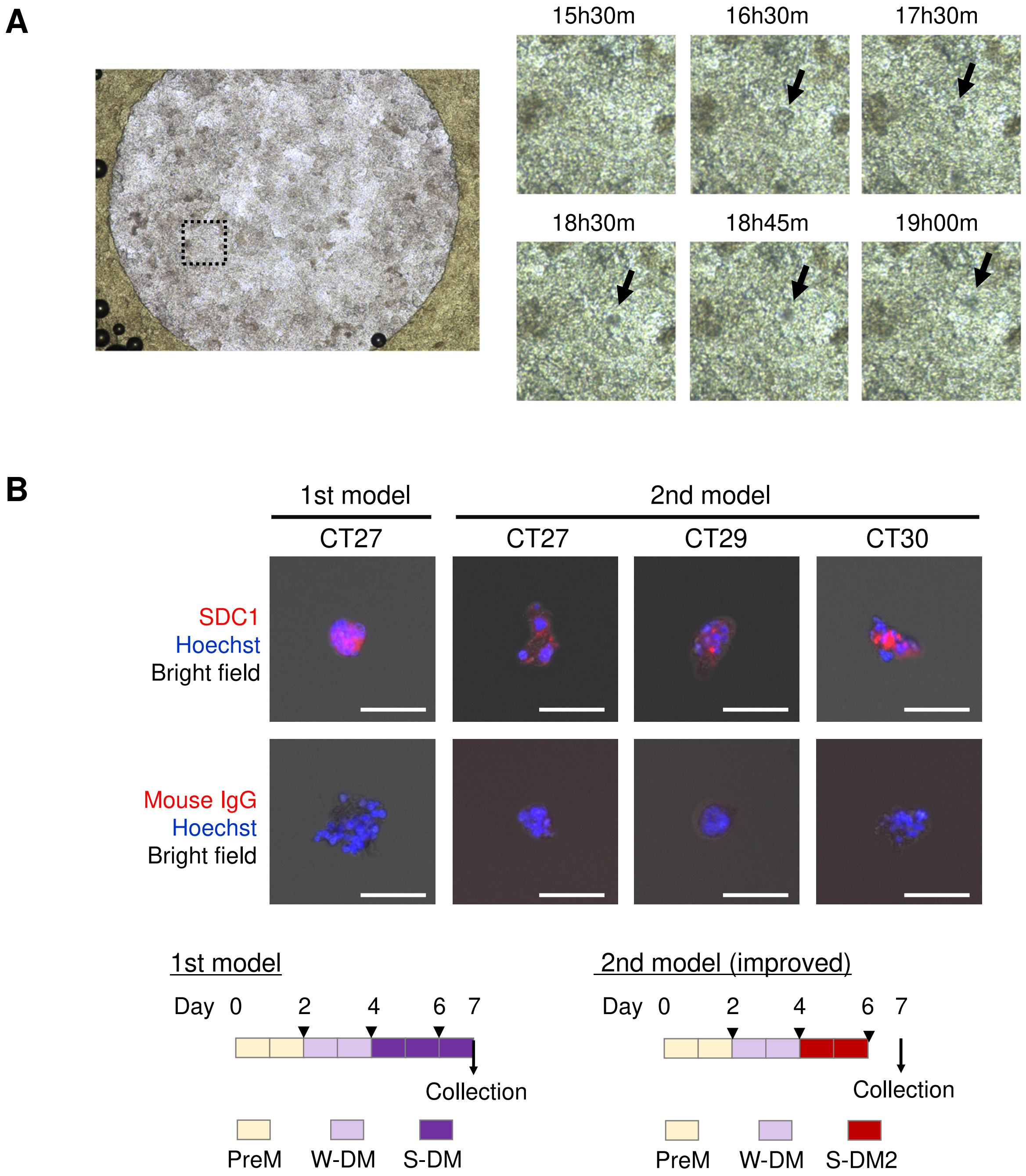

A
15h30m
16h30m
17h30m
18h30m
18h45m
19h00m
B
1st model
2nd model
CT27
CT27
CT29
CT30
SDC1
Hoechst
Bright field
Mouse IgG
Hoechst
Bright field
1st model
Day 0 2 4 6 7
Collection
PreM
W-DM
S-DM
2nd model (improved)
Day 0 2 4 6 7
Collection
PreM
W-DM
S-DM2

Fig. S11. Release of cell aggregates from ST barrier models.

(A) Release of cell aggregates from ST barrier models (1st model). The ST cell barrier model was made with the three kinds of medium shown in Fig. 3D(a). During Day 6 and 7, the surface of the model was monitored by time-lapse imaging.

(B) Immunostaining of SDC1 in cell aggregates from two kinds of ST barrier models (1st and 2nd). Cell aggregates that were released between Day 6 to Day 7 were collected on Day 7. Then, the aggregates were fixed with 4% PFA and subjected to immunostaining with an anti-SDC1 antibody. Images were taken using BZ-X800/810. The scale bars indicate 100 μm.

Fig. S12

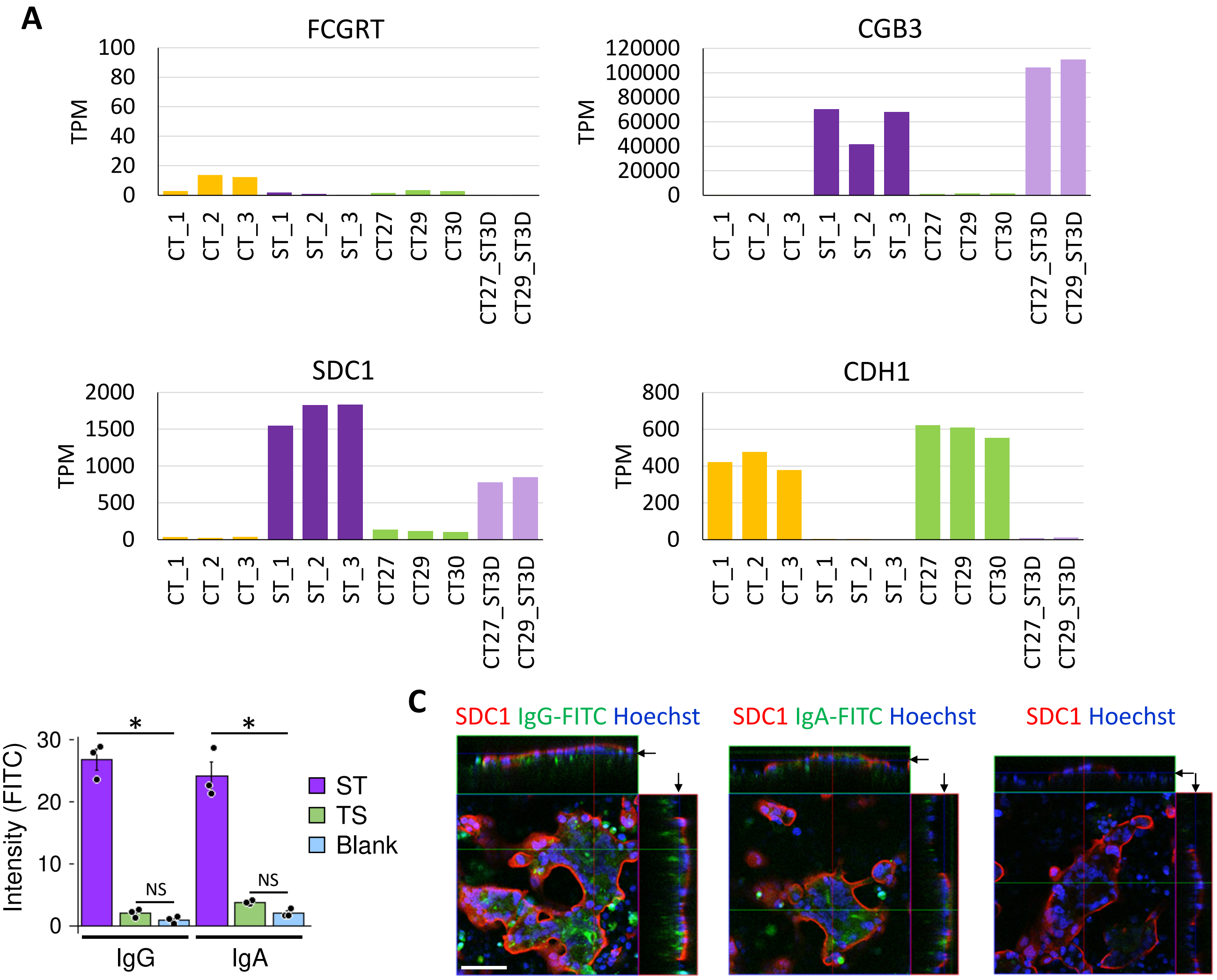
A
FCGRT
TPM
100
80
60
40
20
0
CT_1
CT_2
CT_3
ST_1
ST_2
ST_3
CT27
CT29
CT30
CT27_ST3D
CT29_ST3D
CGB3
120000
100000
80000
60000
40000
20000
SDC1
2000
1500
1000
500
CDH1
800
600
400
200
B
Intensity (FITC)
30
20
10
*
NS
ST
TS
Blank
IgG
IgA
C
SDC1 IgG-FITC Hoechst
SDC1 IgA-FITC Hoechst
SDC1 Hoechst

Fig. S12. Analysis for expression levels of the FCGRT gene and IgG/IgA incorporation .

(A) Gene expression analysis in primary trophoblast cells, TS cells, and TS-derived ST cells. The gene expression levels are from our previous RNA-seq data (Okae H, et al. Cell Stem Cell 22, 50-63 e56, 2018). TPM, transcripts per million. CT, cytotrophoblasts from the 1st-trimester human placenta. ST, syncytiotrophoblasts from the 1st-trimester human placenta. TS, trophoblast stem cells. CT27_ST3D or CT29_ST3D, 3D ST cells differentiated from CT27 or CT29, respectively. The number shown after the name of CT or ST (i.e., CT_1, CT_2, and CT_3, and ST_1, ST_2, and ST_3) mean that samples were isolated from the three placentas. FCGRT is the gene name for FcRn.

(B) Quantitative analysis in fluorescence intensity of FITC-IgG or FITC-IgA in ST models, TS models, and Blank (only a collagen membrane) (N = 3). * $P < 0.05$, Dunnett's test with a condition (Blank) as a control. Data are shown as mean ± standard error (SE).

(C) Confocal microscopy analysis representing incorporation of FITC-IgG or FITC-IgA into ST cells. The scale bar indicates 100 μm.

Fig. S13

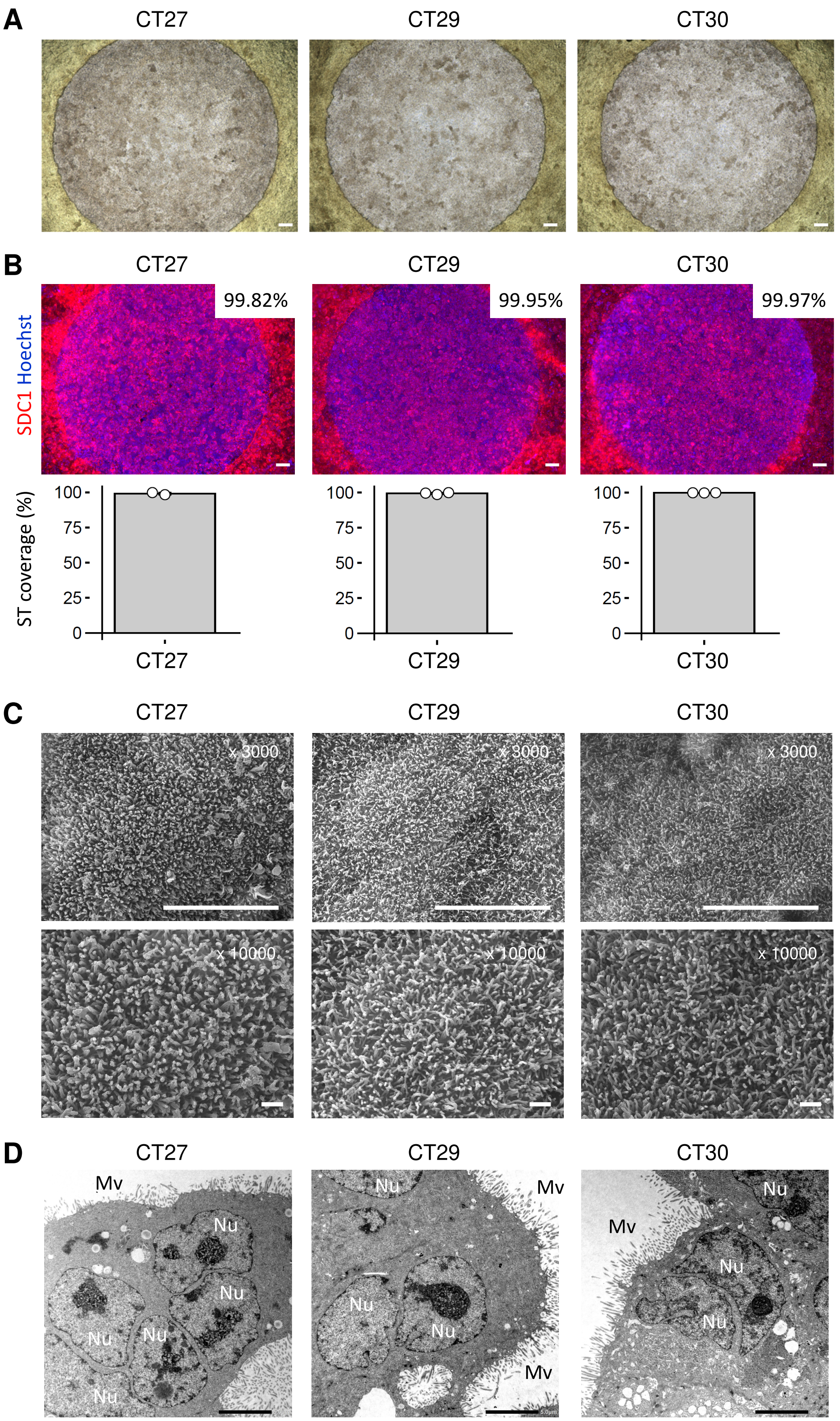
A
CT27
CT29
CT30
B
CT27
CT29
CT30
99.82%
99.95%
99.97%
SDC1 Hoechst
ST coverage (%)
100
75
50
25
0
C
x 3000
x 10000
D
Mv
Nu

Fig. S13. ST barrier models of each TS cell line (CT27, CT29, or CT30)

(A) Phase-contrast microscopic images of ST barrier models that were created according to the 6-day protocol with S-DM2 shown in Fig. 4B.

(B) Immunostaining analysis with antibodies for syndecan 1 (SDC1) was conducted for the barrier models and images were taken using a fluorescent microscope BZ-X800/810. The scale bar indicates 200 μm. The average ST coverages of CT27, CT29, and CT30 were 99.1 ± 0.747% (N=2), 99.4 ± 0.399% (N=3), and 99.9 ± 0.0437% (N=3), respectively (mean ± SE).

(C and D) The surfaces of ST barrier models were analyzed using a scanning electron microscope JSM-7900F (JEOL) (C) and a transmission electron microscope (JEM-1400Flash, JEOL, Japan) (D).

The scale bars indicate 200 μm (A and B), 10 μm (C, x3000), or 1 μm (C, x10000), or 5 μm (D). Images from the barrier models of day 6. Nu, nucleus. Mv, microvilli on the surface of the organoids.

Fig. S14

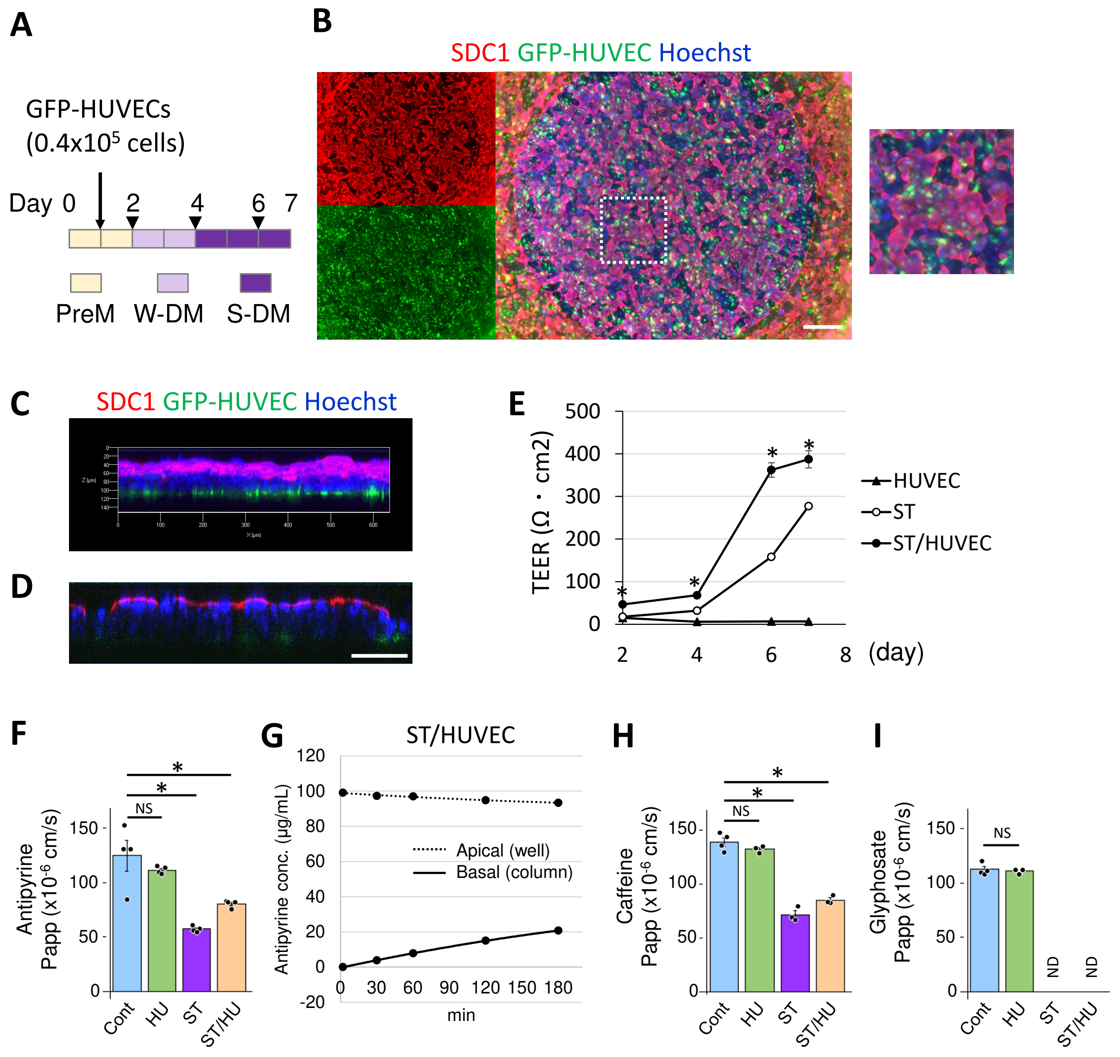
A
GFP-HUVECs
(0.4x10^5 cells)
Day 0 2 4 6 7
PreM W-DM S-DM
B
SDC1 GFP-HUVEC Hoechst
C
SDC1 GFP-HUVEC Hoechst
D
E
TEER (Ω・cm2)
HUVEC
ST
ST/HUVEC
(day)
F
Antipyrine Papp (x10-6 cm/s)
NS
Cont HU ST ST/HU
G
ST/HUVEC
Antipyrine conc. (µg/mL)
Apical (well)
Basal (column)
min
H
Caffeine Papp (x10-6 cm/s)
NS
Cont HU ST ST/HU
I
Glyphosate Papp (x10-6 cm/s)
NS
ND
ND
Cont HU ST ST/HU

Fig. S14. ST/HUVEC co-culture barrier models and translocation of drugs.

(A) The strategy of co-culture for ST/HUVEC models. Triangular marks indicate medium exchange.

(B) Immunostaining of SDC1. The right panel shows a partially magnified image.

(C-D) ST/HUVEC models in a side view by confocal microscopy (C) and a cross-section (D).

(E) TEER measurements in each barrier model (N = 6 for HUVEC or ST/HUVEC, N = 5 for ST).

* $P < 0.05$, Student's *t*-test (ST vs. ST/HUVEC).

(F-I) The permeability of reference compounds in control (Cont, collagen membrane), HUVEC (HU), ST, and ST/HUVEC models [N = 4 (except for HU, ST and ST/HU in the analysis of caffeine, N = 3)].

* $P < 0.05$, Dunnett's test. Data are shown as mean ± SE.

The scale bars indicate 400 μm (B) or 100 μm (D). The values for glyphosate were estimated lower than 0.41 (x 10^{-6} cm/s) by calculation and measurement for the limit of detection. (B-D, F-I) Data were obtained from the barrier models of day 7.

Fig. S15

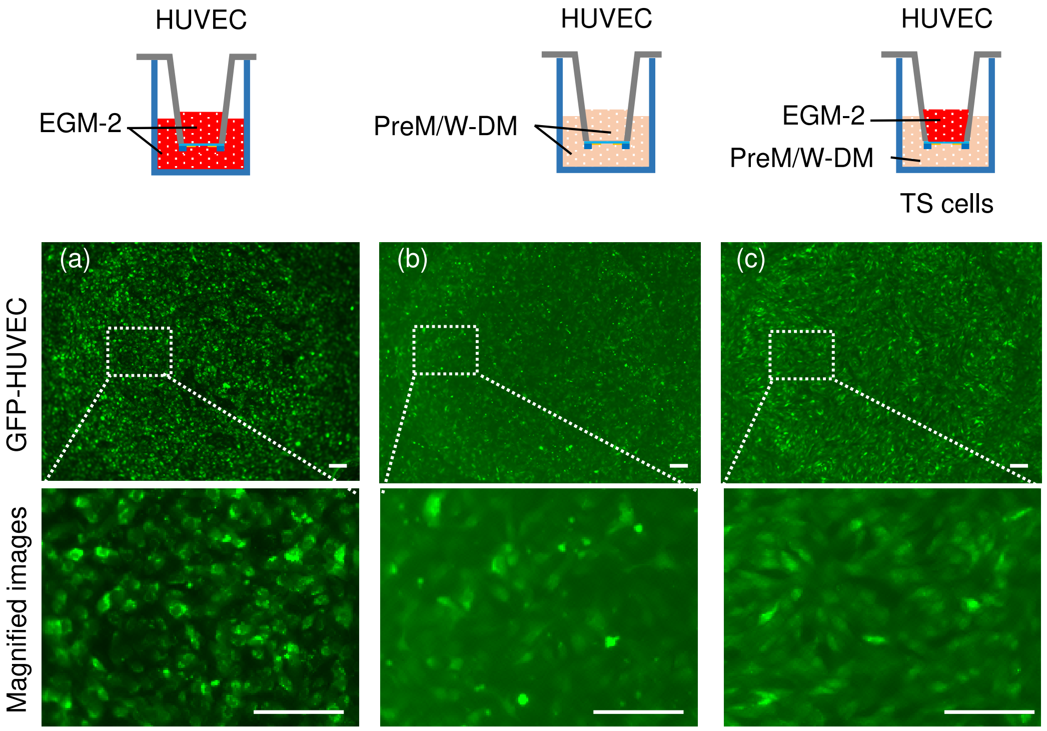
HUVEC
EGM-2
HUVEC
PreM/W-DM
HUVEC
EGM-2
PreM/W-DM
TS cells
(a)
(b)
(c)
GFP-HUVEC
Magnified images

Fig. S15. Morphological change of GFP-HUVECs

(a) GFP-HUVECs were cultured in EGM-2 for 2 days.

(b) GFP-HUVECs were cultured in PreM for 1 day, followed by growth in W-DM for 1 day.

(c) TS cells were seeded onto the bottom surface of the collagen membrane and cultured in PreM for 2 days and then W-DM for 1 day. On day 1, GFP-HUVECs were seeded in the column insert and cultured in EGM-2 for 2 days.

EGM-2, endothelial cell growth medium 2 (Lonza); PreM, pre-culture medium; W-DM, weak differentiation medium. The scale bar indicates 200 μm.

Fig. S16

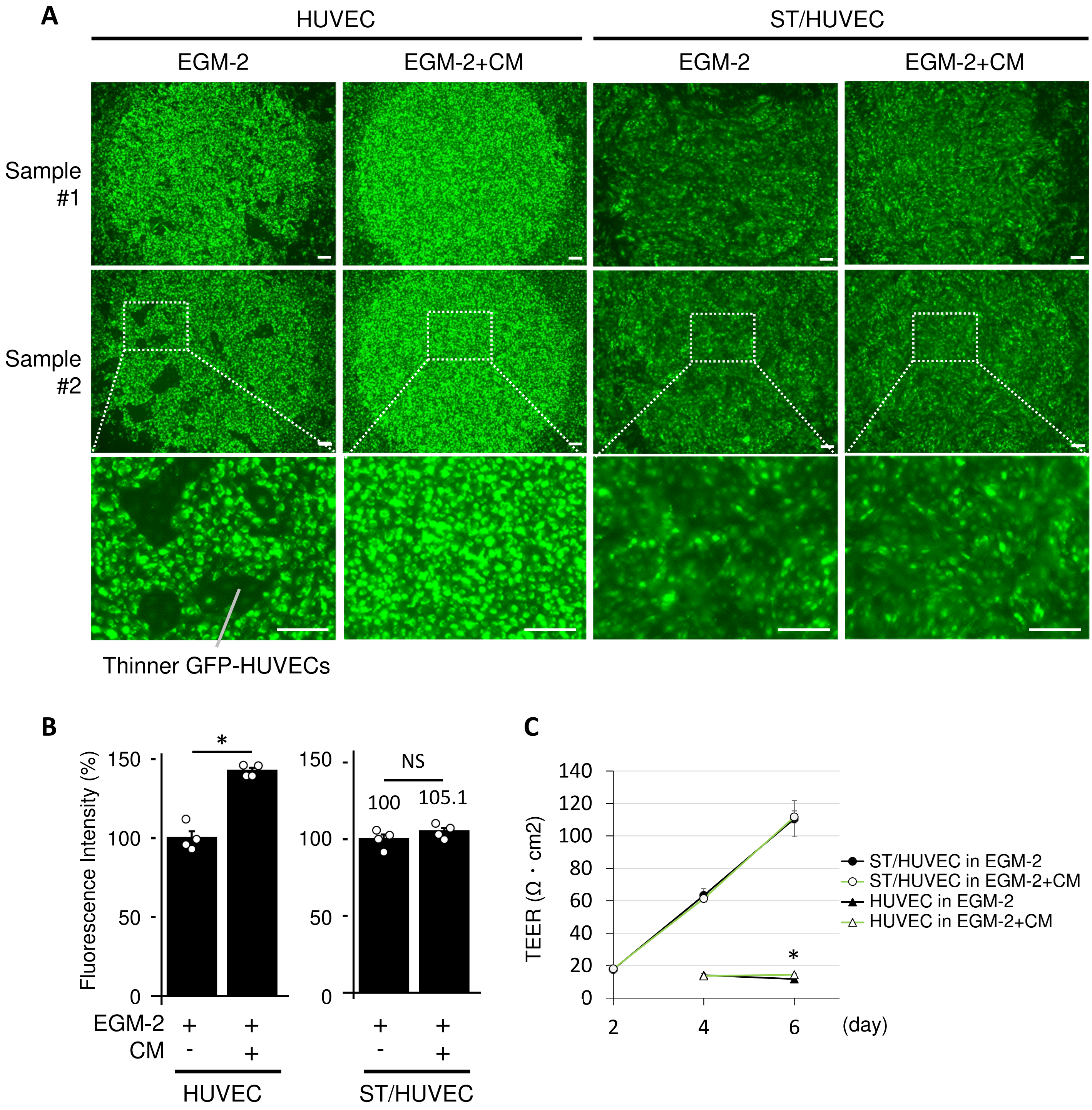

A
HUVEC
ST/HUVEC
EGM-2
EGM-2+CM
EGM-2
EGM-2+CM
Sample #1
Sample #2
Thinner GFP-HUVECs
B
Fluorescence Intensity (%)
150
100
50
0
*
NS
100
105.1
EGM-2
CM
HUVEC
ST/HUVEC
C
TEER (Ω · cm2)
140
120
100
80
60
40
20
0
2
4
6
(day)
*
ST/HUVEC in EGM-2
ST/HUVEC in EGM-2+CM
HUVEC in EGM-2
HUVEC in EGM-2+CM

Fig. S16. Consideration of culture media for culturing GFP-HUVECs

(A) Representative two images of GFP-HUVECs in each group. ST/HUVEC barrier models were generated along with the 6-day protocol shown in Fig. 5A. TS cells were seeded to the collagen membrane on the column-type device and started to culture in PreM. On day 2, culture media in wells were replaced with W-DM, and GFP-HUVECs were added into each insert column, which contains 100 µL of EGM-2 or CM (conditioned medium), at 0.4 x 10^6 cells/mL x 100 µL/column. On day 4, culture media in wells were changed to S-DM2, and those in each column were changed to the corresponding media. On day 6, cells were fixed with 4% PFA and imaged using a fluorescent microscope BZ-X800/810. CM was obtained from normal human lung fibroblasts (NHLF). Briefly, NHLF were cultured with D-MEM high glucose (Fujifilm Wako) containing 10% FBS, 100 units/mL penicillin, and 100 µg/mL streptomycin in a T75 flask. After cells reached 80-90% confluency, the culture medium was replaced with EGM-2. After three days, the medium was collected and centrifuged at 420 x g for 3 min. The supernatant was filtrated (pore size, 0.22 µm), and the obtained medium was stored as CM at -20ºC until use.

(B) Fluorescence intensity of GFP-HUVECs for each group was analyzed using ImageJ version 1.47t (N=4).

(C) TEER measurements in each barrier model (N = 4). * $P < 0.05$, Student's t-test (EGM-2 vs. EGM-2+CM).

CM, conditioned medium; EGM-2, endothelial cell growth medium 2 (Lonza); PreM, pre-culture medium; W-DM, weak differentiation medium. S-DM2, strong differentiation medium 2 containing 2 µM forskolin. The scale bar indicates 200 µm.
