# Supplementary material for: Trophoblast stem cell-based organoid models of the human placental barrier
Source: Nat Commun. 2024 Feb 8;15:962. doi: 10.1038/s41467-024-45279-y (PMC10853531; doi:10.1038/s41467-024-45279-y)
Supplement: Supplementary file 3 — Description of Additional Supplementary Files [file 41467_2024_45279_MOESM3_ESM.pdf]

Description of Additional Supplementary Files

File Name: Supplementary Data 1
Description: List of primers used for the vector construction.

File Name: Supplementary Data 2
Description: Multiple reaction monitoring (MRM) parameters for the target analytes.
