# Supplementary material for: Trophoblast stem cell-based organoid models of the human placental barrier
Source: Nat Commun. 2024 Feb 8;15:962. doi: 10.1038/s41467-024-45279-y (PMC10853531; doi:10.1038/s41467-024-45279-y)
Supplement: Supplementary file 6 — Reporting Summary [file 41467_2024_45279_MOESM6_ESM.pdf]

nature portfolio

Corresponding author(s): Hirokazu Kaji

Last updated by author(s): Dec 25, 2023

Reporting Summary

Nature Portfolio wishes to improve the reproducibility of the work that we publish. This form provides structure for consistency and transparency in reporting. For further information on Nature Portfolio policies, see our Editorial Policies and the Editorial Policy Checklist.

Statistics

For all statistical analyses, confirm that the following items are present in the figure legend, table legend, main text, or Methods section.

n/a	Confirmed	
☐	☒	The exact sample size (n) for each experimental group/condition, given as a discrete number and unit of measurement
☐	☒	A statement on whether measurements were taken from distinct samples or whether the same sample was measured repeatedly
☐	☒	The statistical test(s) used AND whether they are one- or two-sided<br>*Only common tests should be described solely by name; describe more complex techniques in the Methods section.*
☐	☒	A description of all covariates tested
☐	☒	A description of any assumptions or corrections, such as tests of normality and adjustment for multiple comparisons
☐	☒	A full description of the statistical parameters including central tendency (e.g. means) or other basic estimates (e.g. regression coefficient) AND variation (e.g. standard deviation) or associated estimates of uncertainty (e.g. confidence intervals)
☐	☒	For null hypothesis testing, the test statistic (e.g. F, t, r) with confidence intervals, effect sizes, degrees of freedom and P value noted<br>*Give P values as exact values whenever suitable.*
☒	☐	For Bayesian analysis, information on the choice of priors and Markov chain Monte Carlo settings
☒	☐	For hierarchical and complex designs, identification of the appropriate level for tests and full reporting of outcomes
☒	☐	Estimates of effect sizes (e.g. Cohen's d, Pearson's r), indicating how they were calculated

Our web collection on statistics for biologists contains articles on many of the points above.

Software and code

Policy information about availability of computer code

Data collection	*Provide a description of all commercial, open source and custom code used to collect the data in this study, specifying the version used OR state that no software was used.*
Data analysis	StatView version 5.0.1<br>R version 4.3.0 (2023-04-21)<br>ImageJ 1.47t

For manuscripts utilizing custom algorithms or software that are central to the research but not yet described in published literature, software must be made available to editors and reviewers. We strongly encourage code deposition in a community repository (e.g. GitHub). See the Nature Portfolio guidelines for submitting code & software for further information.

Data

Policy information about availability of data

All manuscripts must include a data availability statement. This statement should provide the following information, where applicable:

- Accession codes, unique identifiers, or web links for publicly available datasets
- A description of any restrictions on data availability
- For clinical datasets or third party data, please ensure that the statement adheres to our policy

RNA sequencing data reported in this paper were obtained from our previous study (H. Okae, et al., Cell Stem Cell 22 (1), 50-63 e56, 2018) (Japanese Genotype-

phenotype Archive (JGA) under the accession number: JGA000117[https://humandbs.biosciencedbc.jp/en/hum0086-v2] and JGA000122[https://humandbs.biosciencedbc.jp/en/hum0112-v1]). Data of Figs. 3E, 3K, 3L, 4G, 4I, 4J, 5F, 5G, S4, S12A, S12B, S15B, S15C, S16E, S16F, S16G, S16H, and S16I are provided as a Source Data file.

Research involving human participants, their data, or biological material

Policy information about studies with human participants or human data. See also policy information about sex, gender (identity/presentation), and sexual orientation and race, ethnicity and racism.

Reporting on sex and gender

Use the terms sex (biological attribute) and gender (shaped by social and cultural circumstances) carefully in order to avoid confusing both terms. Indicate if findings apply to only one sex or gender; describe whether sex and gender were considered in study design; whether sex and/or gender was determined based on self-reporting or assigned and methods used.
Provide in the source data disaggregated sex and gender data, where this information has been collected, and if consent has been obtained for sharing of individual-level data; provide overall numbers in this Reporting Summary. Please state if this information has not been collected.
Report sex- and gender-based analyses where performed, justify reasons for lack of sex- and gender-based analysis.

Reporting on race, ethnicity, or other socially relevant groupings

Please specify the socially constructed or socially relevant categorization variable(s) used in your manuscript and explain why they were used. Please note that such variables should not be used as proxies for other socially constructed/relevant variables (for example, race or ethnicity should not be used as a proxy for socioeconomic status).
Provide clear definitions of the relevant terms used, how they were provided (by the participants/respondents, the researchers, or third parties), and the method(s) used to classify people into the different categories (e.g. self-report, census or administrative data, social media data, etc.)
Please provide details about how you controlled for confounding variables in your analyses.

Population characteristics

Describe the covariate-relevant population characteristics of the human research participants (e.g. age, genotypic information, past and current diagnosis and treatment categories). If you filled out the behavioural & social sciences study design questions and have nothing to add here, write "See above."

Recruitment

Describe how participants were recruited. Outline any potential self-selection bias or other biases that may be present and how these are likely to impact results.

Ethics oversight

Identify the organization(s) that approved the study protocol.

Note that full information on the approval of the study protocol must also be provided in the manuscript.

Field-specific reporting

Please select the one below that is the best fit for your research. If you are not sure, read the appropriate sections before making your selection.

☒ Life sciences ☐ Behavioural & social sciences ☐ Ecological, evolutionary & environmental sciences

For a reference copy of the document with all sections, see nature.com/documents/nr-reporting-summary-flat.pdf

Life sciences study design

All studies must disclose on these points even when the disclosure is negative.

Sample size

No statistical methods were applied to predetermine the experimental sample size. The sample size was determined based on previously studied with similar experiments and analyses in the literature (H. Okae, et al., Cell Stem Cell 22 (1), 50-63 e56 (2018), and N. Kobayashi, et al., Nat Commun 13 (1), 3071 (2022)). Sample sizes for all data are indicated clearly in the Methods and Figure legends.

Data exclusions

No data were excluded from the analyses.

Replication

Three cell lines of human trophoblast stem cells (CT27, CT29, and CT30) were used in the present study, and the results indicated successful reproducibility. The number of independent biological replicates for all experiment are indicated clearly in the sections of Methods and Figure legends.

Randomization

Each sample group was compared with no randomization. Randomization was not applicable because defined sample sets were compared.

Blinding

Each experiment was conducted with no blinding because the same investigators designed and conducted the experiments, including the appropriate controls.

Reporting for specific materials, systems and methods

We require information from authors about some types of materials, experimental systems and methods used in many studies. Here, indicate whether each material, system or method listed is relevant to your study. If you are not sure if a list item applies to your research, read the appropriate section before selecting a response.

Materials & experimental systems

n/a	Involved in the study	
☐	☒	Antibodies
☐	☒	Eukaryotic cell lines
☒	☐	Palaeontology and archaeology
☒	☐	Animals and other organisms
☒	☐	Clinical data
☒	☐	Dual use research of concern
☒	☐	Plants

Methods

n/a	Involved in the study	
☒	☐	ChIP-seq
☒	☐	Flow cytometry
☒	☐	MRI-based neuroimaging

Antibodies

Antibodies used

All antibodies used in the study were obtained from manufacturers.
Anti-CDH1 (Cell Signaling Technology, Cat#3195, 24E10, 1:250-1:500 for Immunostaining)
Anti-CDH1 (Takara, Cat#M126, Clone SHE78-7, 1:800 for Immunostaining)
Anti-hCG (Dako, Cat#IR508, 1:10 for Immunostaining)
Anti-GATA3 (Cell Signaling Technology, Cat#5852, 1:200 for Immunostaining)
PE-conjugated anti-SDC1 (Miltenyi Biotec, Cat#130-119-928, Clone 44F9, 1:500 for Immunostaining)
Anti-TP63 (Abcam, Cat#ab124762, 1:400 for Immunostaining)
Anti-Ki-67 (Dako, Cat#M7240, 1:200 for Immunostaining)
Rabbit IgG Isotype control (Cell Signaling Technology, Cat#3900, 1 μg/mL for Immunostaining)
Mouse IgG Isotype control (Cell Signaling Technology, Cat#5415, 1 μg/mL for Immunostaining)
Alexa Fluor 488 conjugated anti-rabbit IgG (Cell Signaling Technology, Cat#4412, 1:400 for Immunostaining)
Alexa Fluor 555 conjugated anti-mouse IgG (Cell Signaling Technology, Cat#4409, 1:400 for Immunostaining)
Alexa Fluor 647 conjugated anti-rabbit IgG (Cell Signaling Technology, Cat#4414, 1:400 for Immunostaining)

Validation

All antibodies were validated by their manufacturers for the applications and species used in this study. Validation statements and literature citations are available on the manufacturer's websites.
Anti-CDH1 (Cell Signaling Technology, Cat#3195, 24E10)
https://www.cellsignal.com/products/primary-antibodies/e-cadherin-24e10-rabbit-mab/3195
Anti-CDH1 (Takara, Cat#M126, Clone SHE78-7)
https://catalog.takara-bio.co.jp/PDFS/m126_ds_j.pdf
Anti-hCG (Dako, Cat#IR508)
https://www.citeab.com/antibodies/3383167-ir508-chorionic-gonadotropin-hcg
Anti-GATA3 (Cell Signaling Technology, Cat#5852)
https://www.cellsignal.jp/products/primary-antibodies/gata-3-d13c9-xp-rabbit-mab/5852
PE-conjugated anti-SDC1 (Miltenyi Biotec, Cat#130-119-928, Clone 44F9)
https://static.miltenyibiotec.com/asset/150655405641/document_6ug6ch8um96736l7glf3tbme2u?content-disposition=inline
Anti-TP63 (Abcam, Cat#ab124762)
https://www.abcam.com/products/primary-antibodies/p63-antibody-epr5701-ab124762.html
Anti-Ki-67 (Dako, Cat#M7240)
https://www.agilent.com/en/product/immunohistochemistry/antibodies-controls/primary-antibodies/ki-67-antigen-(concentrate)-76646
Rabbit IgG Isotype control (Cell Signaling Technology, Cat#3900)
https://www.cellsignal.jp/products/primary-antibodies/rabbit-da1e-mab-igg-xp-isotype-control/3900
Mouse IgG Isotype control (Cell Signaling Technology, Cat#5415)
https://www.cellsignal.jp/products/primary-antibodies/mouse-g3a1-mab-igg1-isotype-control/5415
Alexa Fluor 488 conjugated anti-rabbit IgG (Cell Signaling Technology, Cat#4412)
https://www.cellsignal.jp/products/secondary-antibodies/anti-rabbit-igg-h-l-f-ab-2-fragment-alexa-fluor-488-conjugate/4412
Alexa Fluor 555 conjugated anti-mouse IgG (Cell Signaling Technology, Cat#4409)
https://www.cellsignal.jp/products/secondary-antibodies/anti-mouse-igg-h-l-f-ab-2-fragment-alexa-fluor-555-conjugate/4409
Alexa Fluor 647 conjugated anti-rabbit IgG (Cell Signaling Technology, Cat#4414)
https://www.cellsignal.com/products/secondary-antibodies/anti-rabbit-igg-h-l-f-ab-2-fragment-alexa-fluor-647-conjugate/4414

Eukaryotic cell lines

Policy information about cell lines and Sex and Gender in Research

Cell line source(s)

Human trophoblast stem cell lines (CT27, CT29, and CT30) were established in our previous study (H. Okae, et al., Cell Stem Cell 22 (1), 50-63 e56 (2018)).

Authentication

Human trophoblast stem cell lines (CT27, CT29, and CT30) were derived and authenticated by Takahiro Arima and Hiroaki Okae (Tohoku University Graduate School of Medicine, Sendai, Japan) (H. Okae, et al., Cell Stem Cell 22 (1), 50-63 e56 (2018)).

Mycoplasma contamination

All cell lines were tested negative for mycoplasma contamination.

Commonly misidentified lines
(See ICLAC register)

No commonly misidentified cell line was used in this study.
